# Inactivation of Norovirus on Dry Copper Alloy Surfaces

**DOI:** 10.1371/journal.pone.0075017

**Published:** 2013-09-09

**Authors:** Sarah L. Warnes, C. William Keevil

**Affiliations:** Centre for Biological Sciences, University of Southampton, Southampton, United Kingdom; University of Maryland, United States of America

## Abstract

Noroviruses (family *Caliciviridae*) are the primary cause of viral gastroenteritis worldwide. The virus is highly infectious and touching contaminated surfaces can contribute to infection spread. Although the virus was identified over 40 years ago the lack of methods to assess infectivity has hampered the study of the human pathogen. Recently the murine virus, MNV-1, has successfully been used as a close surrogate. Copper alloys have previously been shown to be effective antimicrobial surfaces against a range of bacteria and fungi. We now report rapid inactivation of murine norovirus on alloys, containing over 60% copper, at room temperature but no reduction of infectivity on stainless steel dry surfaces in simulated wet fomite and dry touch contamination. The rate of inactivation was initially very rapid and proportional to copper content of alloy tested. Viral inactivation was not as rapid on brass as previously observed for bacteria but copper-nickel alloy was very effective. The use of chelators and quenchers of reactive oxygen species (ROS) determined that Cu(II) and especially Cu(I) ions are still the primary effectors of toxicity but quenching superoxide and hydroxyl radicals did not confer protection. This suggests Fenton generation of ROS is not important for the inactivation mechanism. One of the targets of copper toxicity was the viral genome and a reduced copy number of the gene for a viral encoded protein, VPg (viral-protein-genome-linked), which is essential for infectivity, was observed following contact with copper and brass dry surfaces. The use of antimicrobial surfaces containing copper in high risk closed environments such as cruise ships and care facilities could help to reduce the spread of this highly infectious and costly pathogen.

## Introduction

Gastroenteritis is a major cause of morbidity and mortality worldwide and is responsible for approximately 5–8 million deaths per year. It is estimated that norovirus (family *Caliciviridae*) gives rise to more than 267 million infections worldwide per year including 23 million in the US alone. This small, single stranded, positive sense RNA virus is responsible for over 90% cases of non-bacterial and approximately half of all cases of gastroenteritis (reviewed in [1], [2], [3]). Norovirus is now as important as rotavirus as a cause of diarrhoea and vomiting in hospitalised children in some countries [4].

The disease is usually contracted by ingestion of contaminated food, water, person-to-person contact and touching contaminated surfaces [5], [6]. Infection is also transmitted by aerosols and prolonged viral shedding of high virus load, including asymptomatic individuals, increases the risk of infection spread [7]. Norovirus gastroenteritis is self-limiting but extremely infectious with a low infectious dose and is responsible for many outbreaks, often seasonal, especially in closed environments e.g. cruise ships and health-care facilities. Most reported cases are in the under 5 years old but the highest economic costs are in the care of elderly patients in residential care [5]. The disease may be life threatening in severely ill and vulnerable patients and has been linked to Crohn's disease and necrotising enterocolitis in neonates [8].

The virus does not have an envelope, conferring resistance to some cleaning detergents, alcohols, food preservation chemicals; it can survive on surfaces (especially if surfaces are contaminated with detritus and food residues [9] in the environment, and resist a wide pH range and temperatures from −20°C to 72°C [10], [11], [12]. The contribution of contaminated surfaces in the spread of infection has been described previously [3], [6]. In human challenge studies Gerhardts et al. [13] demonstrated a chain of bacterial and viral transfer from a single contaminated individual to surfaces and from there to other personnel with the risk of infection greatest in pathogenic strains with a low infectious dose. Thornley et al. [14] suggested that persistently contaminated fomites resulted in transmission of infection in flight attendants over an 8-day period following a single vomiting incident of a passenger with norovirus. Studies have also shown the transfer of noroviruses from cleaning cloths of varying composition and absorbency to surfaces and *vice versa* and also spread of virus from a single fingertip to up to 7 surfaces [15], [16] perpetuating the spread of infectious virions.

The use of antimicrobial surfaces in clinical and community environments may help to reduce the spread of infection, especially if combined with rigorous and effective cleaning regimes. Laboratory studies have described the rapid death of bacterial, fungal and viral pathogens on copper alloy surfaces [17]–[27] and also prevention of antibiotic resistance horizontal gene transfer between pathogens [27]. The results from these studies led to clinical trials worldwide in clinical and children's facilities where a reduction in microbial bioburden was observed in rooms with copper surfaces [28], [29]. Of great significance, a recent study of 3 US hospital intensive care units has shown more than a 50% reduction in the infection rate when copper alloys have replaced conventional touch surfaces for 6 highly touched objects (bed rails, over-bed tables, chair arm, call button, computer accessories and intravenous poles [30]).

Sensitive detection methods for human norovirus are available, primarily PCR amplification of genes encoding viral capsid or viral RNA dependant RNA polymerase (RdRp) from cDNA [31]. However, there is no correlation between these methods and infectivity [32] and there are no available methods to assess viral infectivity, other than human challenge, because of the absence of suitable tissue culture systems [33]. Therefore research has concentrated on feline or murine surrogates. In this study we have investigated the infectivity of murine norovirus (MNV), the closest phylogenetic surrogate to the human virus, exposed to dry touch copper and copper alloy surfaces, containing at least 60% copper, assessed by plaque assay in mouse macrophage monocyte cell line, RAW 264.7 [34], [35]. Stainless steel was used as a control surface throughout. We investigated the possible roles of Cu (I) and Cu (II) in viral inactivation and their effect on the integrity of the viral genome following contact of the virus with copper surfaces. The norovirus genome consists of a positive strand RNA of approximately 7.5 kb, and replicates in the host cell cytoplasm. There are 4 open reading frames (ORF); ORF 2 and 3 encode for the capsid proteins and a recently discovered ORF 4 [36] produces a protein that, although not essential for infectivity, affects virulence. The production of sub-genomic strand duplicating ORF 2–4 increases the capacity of the relatively small genome. The ORF1 encodes a polyprotein that is cleaved by viral protease, NS6, into several non-structural proteins. One of these, NS5, encodes for VPg (viral-protein-genome-linked), which is essential for infectivity. It binds to 5′ end of the viral genome acting as a primer initiating translation of viral RNA and also as a protein primer for the viral RdRp [37]. We have observed previously the destruction of bacterial plasmid and genomic nucleic acid on copper and copper alloy dry surfaces. In this study we investigated the effect of norovirus exposure to copper surfaces on the entire genome and as a more sensitive and quantitative assay investigated the effect on a single gene i.e. production of VPg using reverse transcriptase quantitative PCR (RT-qPCR).

## Results

### Infectivity of murine norovirus (MNV) is destroyed on copper and copper alloy surfaces but not on stainless steel for simulated wet fomite and dry touch contamination

**Figure 1:**
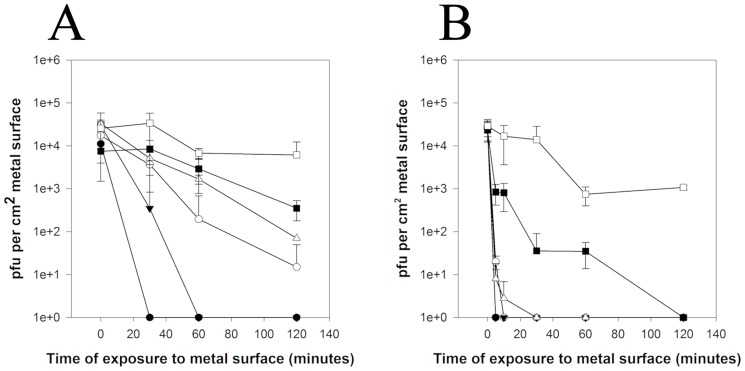
Efficacy of copper alloys to reduce infectivity of wet fomite (A) and dry touch (B) contamination with MNV at room temperature. Plaque assay is described in the text but briefly a dilution series of control and test virus was plated for 60 minutes onto a monolayer of RAW 264.7 cells, then overlaid with agarose and incubated 48–72 hours. Monolayers were stained with vital stain, Neutral Red, and areas of infected and lysed cells can be visualised as plaques and enumerated. Approximately 5×10^4^ pfu were applied to test surfaces (copper (•), phosphor bronze (95% copper) (○), copper nickel (89% copper) (▾), cartridge brass (70% copper) (Δ), nickel silver (65% copper) (▪), stainless steel (□)) in either 20 µL (dries in 30 minutes) or 1 µL (dries in seconds) to represent wet and dry contamination, respectively. No significant loss of infectivity was observed on stainless steel for both types of inocula. Error bars represent ± SD and data are from multiple experiments.

An inoculum of 5×10^4^ pfu MNV applied to copper, and high copper content alloys, phosphor bronze and copper nickel, to simulate wet fomite contamination was rapidly inactivated at room temperature using plaque assay. No infectious virus was evident after 30 minutes on copper and 60 minutes on copper nickel (Figure 1A). There was a 2–4 log reduction for phosphor bronze, cartridge brass and nickel silver respectively after 2 hours at room temperature. Increasing the viral load 50×did not affect kill times (data not shown). There was no significant reduction in infectivity following 2 hours contact with stainless steel at room temperature.

Figure 1B demonstrates that virus inactivation is even more rapid if a ‘dry’ inoculum of virus is used i.e. same size inoculum is applied in very low volume (1 µL) which dries instantly on contact and corresponds to dry touch contamination. All virus is inactivated on copper and copper nickel over the first 5 minutes contact and after 10 and 30 minutes for phosphor bronze and cartridge brass, respectively. Nickel silver, which has the lowest copper content, was inactivated after 2 hours. There was a slight reduction in infectivity on virus exposed to stainless steel, suggesting rapid drying also has an effect.

**Figure 2:**
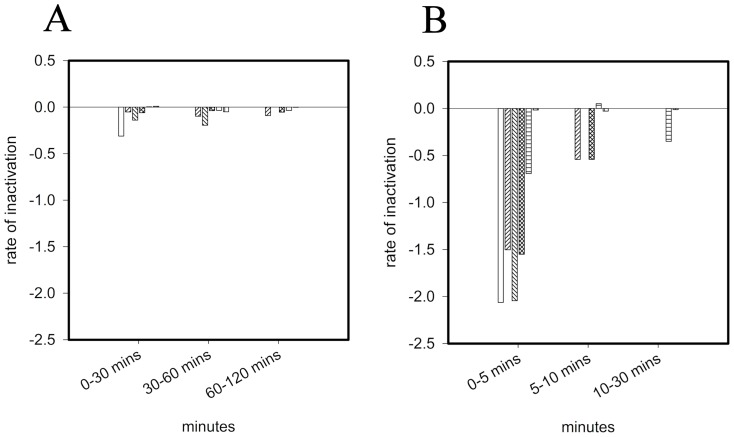
Comparison between inactivation rates of MNV in wet fomite (A) and dry touch (B) contamination on copper surfaces. Inactivation rates were calculated for various contact times of MNV exposed to test surfaces as described in the text (from the results generated in Figure 1). (copper (white bars), phosphor bronze (95% copper) (forward diagonal striped bars), copper nickel (89% copper) (backward diagonal striped bars), cartridge brass (70% copper) (cross hatch bars), nickel silver (65% copper) (horizontal striped bars) and stainless steel (vertical striped bars)). Error bars represent ± SD and data are from multiple experiments.

Calculation of inactivation rate at specific times reveals that the highest rate of MNV inactivation on copper surfaces occurs upon immediate contact (Figure 2). Inactivation was up to 10 times faster in ‘dry’ touch contamination (Figure 2B); on copper rates were −2.06 and −0.3 for dry and wet contamination, respectively (for the initial timepoints). Inactivation rates were proportional to percentage copper: R^2^ =  0.926 (Figure S1 Supporting Information) except that copper nickel (89% copper) was slightly more effective than phosphor bronze (95%). This is now being investigated further with a larger range of copper nickels and also investigating if the surface finish affects the virus inactivation rate.

### Rate of inactivation of MNV on copper surfaces is affected by temperature

**Figure 3:**
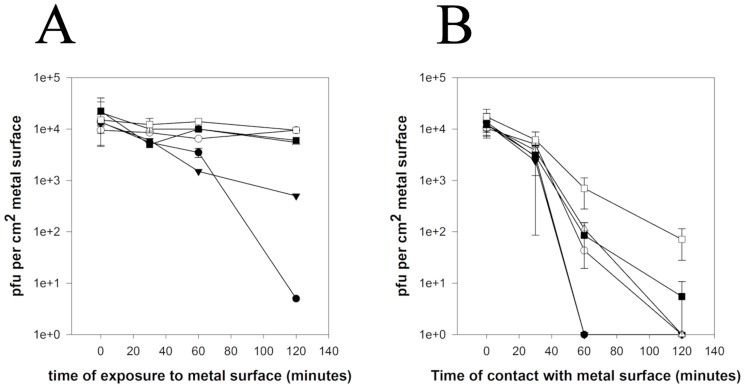
Efficacy of copper alloys to reduce infectivity of wet fomite contamination with MNV at 4°C (A) and 37°C (B). Approximately 5×10^4^ pfu were applied to test surfaces that had been acclimatised to required temperature (copper (•), phosphor bronze (95% copper) (○), copper nickel (▾), cartridge brass (70% copper) (Δ), nickel silver (65% copper) (▪), stainless steel (□)) in 20 µL (‘wet’ inoculum). Virus was removed and assessed for infectivity using plaque assay.

If MNV is inoculated onto surfaces at 4°C inactivation still occurs on copper but at least 4 times more slowly and significant reduction was seen after 2 hours on copper nickel. Little inactivation had occurred on other metals at this time. The inoculum remained wet over the 2 hour testing period (Figure 3A). In contrast at 37°C although for the first 30 minutes of contact there was little reduction of MNV infectivity subsequent inactivation was faster with at least a 3-log reduction on all alloys at 2 hours (Figure 3B). It is unclear if the initial lag is due to any differences between the temperatures of inoculum and the metal. After 2 hours contact with stainless steel at 37°C norovirus was still infectious but there was a considerable reduction in infectivity (2-log) compared to results at room temperature.

### Inactivation of MNV on dry copper surfaces involves copper (II) and especially copper (I) ions but not superoxide or hydroxyl radicals (‘wet’ inoculum)

**Figure 4:**
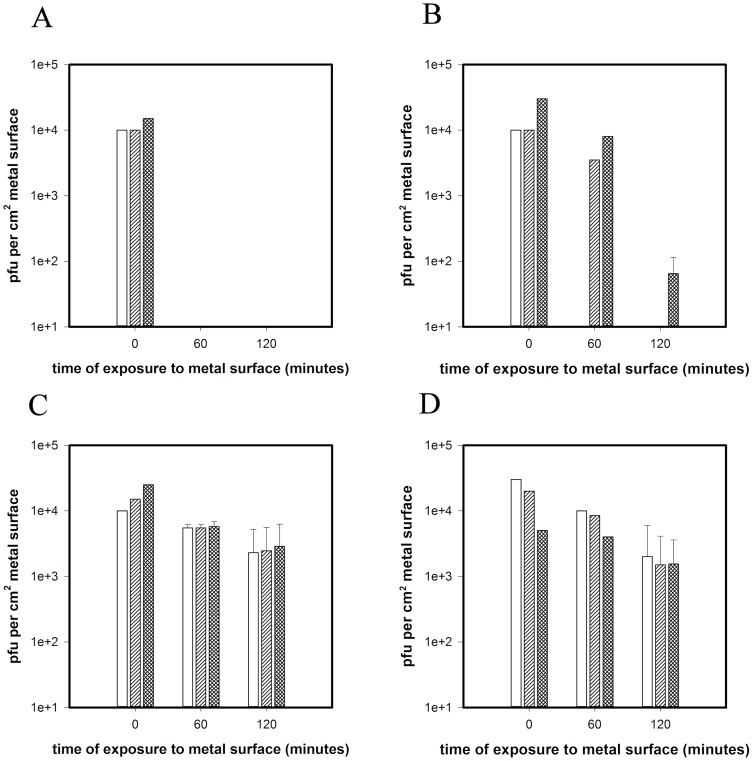
Inactivation of MNV on copper surfaces in the presence of quenchers D-mannitol or Tiron (A) or chelators EDTA or BCS (B) and to remove hydroxyl radical or superoxide, copper II or Cu I, respectively. Approximately 5×10^4^ pfu MNV was inoculated onto metal surfaces in the presence of chelators or quenchers of reactive oxygen species and assessed for infectious virus using plaque assay as described in text. The results were compared to those obtained without chelators or quenchers to ascertain if there was a protective effect. No quenchers or chelators present is represented by white bars; D-mannitol (A) or EDTA (B) represented by diagonal striped bars; Tiron (A) or BCS (B) represented by cross hatched bars. No significant reduction of infectivity occurred in the presence of any quenchers or chelators on stainless steel surfaces (even though D-mannitol has been reported to interfere with HSV replication) (C and D respectively). Error bars represent ± SD and data are from multiple experiments.

Addition of D-mannitol or Tiron, at the same time as virus, to quench hydroxyl radicals or superoxide (Figure 4A striped and cross hatch bars respectively) does not protect MNV from inactivation on copper and the virus is inactivated following 60 minutes contact, the same as virus inoculated without quenchers (Figure 4A white bars). Varying the concentration of the quenchers and addition of superoxide dismutase did not affect results (data not shown). There was no significant loss of infectivity for virus inoculated onto stainless steel compared to virus inoculated with or without quenchers with approximately 10^4^ pfu recovered after 2 hours contact (Figure 4C).

Addition of EDTA at the same time as the virus to chelate Cu(II) was protective for the initial 60 minutes of virus contact with copper (Figure 4B striped bars) but prolonged protection was observed in the presence of BCS (Figure 4B cross hatch bars). This suggests that Cu (I) is important in the inactivation of MNV on dry copper. Inoculation without chelators present resulted in total inactivation by 60 minutes (Figure 4B white bars). There was no significant loss of infectivity in virus inoculated onto stainless steel compared to virus inoculated with or without chelators (Figure 4D).

### Degradation of the entire MNV genome occurs on copper and brass surfaces

**Figure 5:**
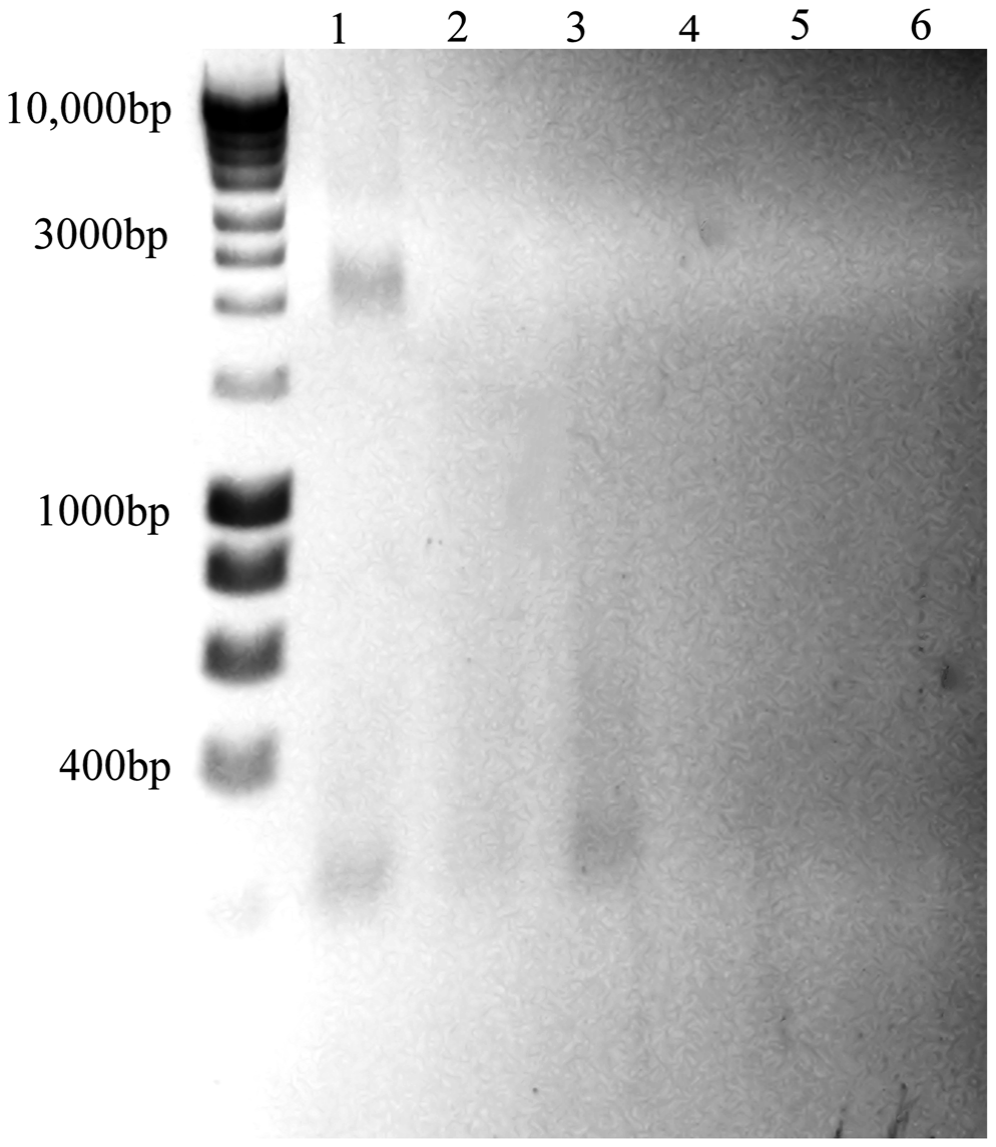
Destruction of entire MNV genome occurs on copper. MNV (PEG concentrate) was exposed to copper (lane 1), cartridge brass (lane 2) or stainless steel (lane 3) for 2 hours. Viral RNA was purified using Qiagen mini prep viral RNA kit and fragments separated on non-denaturing 1% agarose gel electrophoresis and visualised in UV light box. Viral RNA has degraded on copper, less on brass and not at all on stainless steel (see control RNA S2 Supplementary Information). Lanes 4, 5 and 6 are PEG precipitation of uninfected cells (mock) applied to stainless steel, brass and copper respectively. Virus added to all surfaces and removed immediately was similar to lane 1 although some reduction in intensity on copper was visible (not shown). DNA ladder is Bioline hyperladder I (HL1 1 Kb)

PEG concentrated virus was exposed to copper, brass and stainless steel for 2 hours. Virus was removed and total RNA purified and fragments separated by non-denaturing agarose gel electrophoresis (Figure 5). There is a band of native viral RNA from stainless steel that is not visible in samples exposed to copper or cartridge brass. There is some evidence of degraded RNA (less than 300bp) in all three samples. Negative controls were prepared from mock infected cells. MNV used for inoculation but not exposed to metal surfaces was used as a control; purified genomic RNA was analysed by non-denaturing electrophoresis producing band at 2000–2500 bp for native conformation (total size denatured genome is 7382 bp)(Figure S2, Supporting Information)

### MNV exposed to copper and brass surfaces has a lower concentration of viral gene, *NS5*, essential for infectivity because of production of VPg (viral-protein-genome-linked)

**Figure 6:**
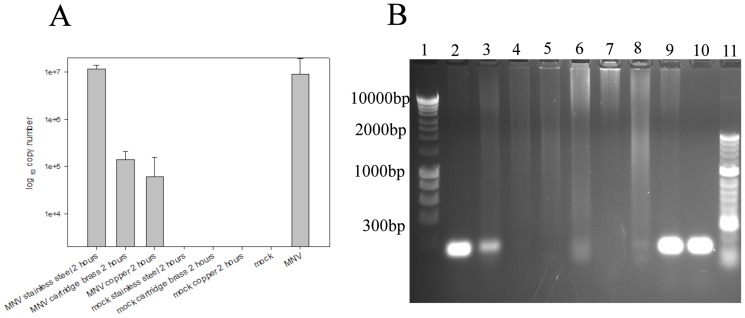
The degradation of viral RNA observed on copper and brass surfaces affects individual genes. cDNA was generated from the RNA of virus recovered from dry surfaces following 2(A). A large copy number is present in virus removed from stainless steel and untreated virus but greatly reduced from brass and copper. This is also evident from electrophoresis of PCR products (B). Lanes 2–4 is virus removed from stainless steel, brass and copper showing reduction in VPg intensity respectively. Lanes 5–7 are mock infected cells. Lane 8 and lane 9 are mock infected cells and infected cell lysate, respectively, that had not been applied to surfaces. Lane 10 is amplified gene from standard cDNA.

The genomic RNA of MNV exposed to copper, brass and stainless steel surfaces was purified and cDNA prepared. qPCR amplification of a 70 bp region of VPg demonstrated a reduction in the copy number of virus removed from copper and brass (Figure 6A). Analysis of the PCR products by electrophoresis showed a reduction in intensity of amplified region that is proportional to percentage copper (Figure 6B). Virus that has been removed from stainless steel is similar to equivalent volume of virus not exposed to surfaces. Virus was also removed from all test surfaces immediately (time 0) and there was no significant difference in copy number from the stainless steel 2 hour sample shown (time 0 samples not shown).

## Discussion

The human cost and also the economic burden of norovirus infection is a huge problem worldwide. In the UK, norovirus costs the National Health Service at least £100 million/year, in times of high incidence, and up to 3000 people admitted to hospital per year in England. The incidence in the community is thought to be about 16.5% of the 17 million cases of infectious intestinal disease, in England per year and there is evidence that this burden is increasing [38]. In the US the costs escalate to more than $2 billion per year on outbreaks with endemic costs of more than $500 million per year (reviewed in [1]). The low infectious dose, prolonged survival at a range of temperatures and humidity for up to 7 days on dry inanimate surfaces, resistance to commonly used disinfectants, long infectious period and prolonged shedding all increase the risk of disease spread. Approximately 30% of infections from norovirus are asymptomatic and the virus can be transmitted although at a lower frequency compared to symptomatic individuals who can shed up to 10^10^ copies of viral RNA per gram faeces [39]. Lopman, et al. [5] suggested the highest risk in transmission of infectious norovirus is initially, over the first 24 hours, from direct contact but subsequent contamination of the environment produces a risk that lasts much longer, for at least 2 weeks. In addition, direct hand contact or even cleaning cloths used to wipe contaminated surfaces can spread infectious virus to other environmental surfaces. Because norovirus is resistant to many commonly used cleaning agents it is necessary to use 0.1% hypochlorite (equivalent to 1000ppm chlorine) to disinfect surfaces. However, regular use of this biociode bleaches and degrades a range of commonly used touch surface materials and is hazardous in poorly ventilated areas [40].

In this study we have shown that murine norovirus is also rapidly inactivated on copper surfaces. A significant reduction occurred on copper, cartridge brass and nickel silver after 2 hours at room temperature. The rate of inactivation was approximately proportional to copper content but further studies are required to evaluate the effect of different metal surface finishes and the copper ion release rate from individual alloys. In dry touch contamination viral inactivation is extremely rapid, with the highest rate of inactivation occurring in the first 5 minutes. The process appears to be a result of a combination of copper action and drying process followed by a slower rate of inactivation; Li and Dennehy [41] observed that bacteriophages also lost infectivity on copper but as soon as the inoculum dried no further inactivation occurred and Abad et al. [42] observed that the drying process affected persistence of poliovirus and adenovirus on environmental fomites. Sharps et al. [43] observed that virus retained infectivity in transfer ‘wet’ fomite from stainless steel to fingertips and fruits but transfer was reduced if contaminating inoculum was allowed to dry. We have found that the rate of inactivation is also affected by temperature: inactivation occurs more slowly at 4°C and is faster over a 2 hour period at 37°C. It is unclear if the mechanism of copper inactivation of norovirus is different in wet or dry scenarios. The clear stages of inactivation may reflect populations of virions at various stages of replication (e.g. complete or incomplete capsids and packaging) with varying susceptibilities and rates of inactivation.

We have previously shown that although dry copper surfaces are efficacious against a range of bacteria the copper killing mechanism is different. In Gram-negative cells the outer membrane is the initial target and Fenton reactions between respiration generated oxygen radicals and copper ions results in generation of reactive oxygen species causing the cells to commit ‘metabolic suicide’ [25]. In this current study we have observed that Cu(II) was important in the short term but it is Cu(I) that is the primary effector of copper surface inactivation of norovirus. Shionoiri et al. [44] observed Cu(I) was important in inactivation of feline norovirus (FCV), but they investigated copper iodide nanoparticles in solution and Sagripanti et al. [45] discovered that superoxide was not involved in the destruction of the double stranded DNA of Herpes Simplex Virus on copper and only partial protection was seen for hydroxyl radicals. We also found that Cu(I) release on contact surfaces did not result in the generation of hydroxyl radicals or superoxide indicating Fenton chemistry is not important. This suggests that copper ions are having direct effect in virus inactivation. Copper ions have been observed to cause aggregation of virus particles [46].

We investigated if the viral RNA was affected by the copper because virus inactivation is possible without obvious effects on the genome [31]. The entire RNA genome was destroyed on copper suggesting the function of other genes could also be affected so the next step was to investigate if individual genes are affected by more sensitive and quantitative molecular methods. Norovirus replication is rapid and efficient because the positive strand genome does not need a DNA stage, produces its own primer in VPg and a viral RNA polymerase and occurs within the cytoplasm without the need to traverse the cell's nuclear membrane. Exposure to copper and brass resulted in a reduction in the copy number of VPg gene that was unaffected on stainless steel. The extent of RNA destruction was proportional to the percentage of copper. Investigations into the effect on other genes and the viral capsid are now underway to determine the sequence of events as norovirus inactivates on copper surfaces and actual targets.

Recombination events resulting from small amino acid substitutions affecting antigenic domains (P2) and in the viral polymerase have led to the evolution of more virulent norovirus strains. GII.g/GII.12 was first isolated in Australia 2008 and results in an increased severity of disease that is not restricted to individuals with specific blood groups, unlike earlier strains, and is capable of zoonotic transmission [1], [47]. The survival of infectious norovirus for long periods on surfaces and foods may contribute to interspecies transmission and the evolution of more virulent strains. The destruction of the viral genome we have observed on copper surfaces may mitigate against this as well as prevent the spread of infection.

There is now a considerable body of evidence from laboratory based studies that copper alloys are efficacious against a diverse range of pathogenic microorganisms. Earlier studies demonstrated a rapid kill of *Escherichia coli* O157 [17], [19], [26], *Listeria monocytogenes* [20] and methicillin-resistant *Staphylococcus aureus* (MRSA) [18] which evolved from commensals into a serious threat to world health. This was followed by observations that both vegetative cells and spores of virulent toxin producing *Clostridium difficile*, responsible for numerous hospital acquired infections (HAI), were also destroyed on copper [22]. The increased antimicrobial therapies required to combat MRSA has resulted in the evolution of potentially more serious multi-drug resistant bacterial pathogens including vancomycin-resistant enterococci and more recently the rise in serious, difficult to treat infections by Gram-negative *Enterobacteriaceae* producing extended spectrum β-lactamases and metallo-β-lactamases, including NDM-1(New Delhi metallo-β-lactamase) which is resistant to all β lactams. This is often located on plasmids containing many other resistance and virulence genes. However, all these emerging pathogens are destroyed on copper and copper alloy surfaces although we now know the killing mechanism is not universal [24]–[27]. We also observed in a previous study the rapid transfer of *bla*_NDM-1_ to other bacterial contaminants on stainless steel surfaces which did not occur on copper [27]. Therefore copper surfaces could also help to prevent horizontal gene transfer (HGT) which is ultimately responsible for the spread in resistance to our existing antibiotics. We have now shown that MNV is also rendered non-infectious on dry copper alloy surfaces and we are currently investigating efficacy against a range of respiratory viral pathogens following an earlier study on influenza A [21].

There have been numerous clinical trials following encouraging results from laboratory studies [28], [29]. The recent report by Salgado et al. [30] based on trials at 3 hospitals is extremely encouraging, that replacing only 6 items within a hospital ICU room could have such an impact on reducing the infection rate by more than 50%. This suggests that copper alloy surfaces may also be usefully employed in other high risk areas such as care homes, public transport and even in the home.

The use of copper alloy dry surfaces in health care and community environments could be invaluable in preventing the spread of bacterial, fungal and viral pathogens, including norovirus, that contaminate dry surfaces and perpetuate the infection cycle. The race to develop effective antimicrobials against pathogens that have evolved mechanisms to evade our existing ones is fierce and led to a fear that we are entering a pre-antibiotic era.

Copper alloys, although they provide a constant killing surface, should always be used in conjunction with regular and efficient cleaning and decontamination regimes using non-chelating reagents that could inhibit the copper ion activity.

## Materials and Methods

### Viral strains and cell lines

Murine norovirus 1, MNV-1, CW1, and the mouse monocyte macrophage line, RAW 264.7, were supplied by Professor Herbert Virgin IV, Washington University, US. The semi-adherent cell line was maintained at sub-confluence to prevent loss of characteristic phenotype and maintained in HEPES buffered Dulbecco's Modified Eagle Medium (DMEM) containing GlutaMAX, 25 mM D-glucose, 10% foetal bovine serum and without sodium pyruvate at 37°C in the presence of 5% CO_2_. The cells adhere to tissue culture grade plastic through cation- dependant and independent receptors but can easily be removed by scraping.

To ensure at least 99% cells were infected virus stocks were prepared by infecting cells with multiplicity of infection of approximately 5. The inoculum was removed after 90 minutes incubation at 37°C in the presence of 5% CO_2_, replaced with fresh medium and incubated for a further 48 hours or until characteristic cytopathic effect (cpe) was observed. Infected cells were exposed to 3 freeze/thaw cycles, cell debris was removed by low speed centrifugation and supernatant stored at −80°C. Conventional ultrapurification methods may affect structure and infectivity of murine norovirus so a further purification step was performed using polyethylene glycol(PEG) and NaCl precipitation (BioVision Inc, US) which concentrated the sample 100 times. Infected cell supernatants and PEG precipitated virus were used in infectivity assay, Mock infected cells were used as controls

### Preparation of sample surfaces

Metal coupons (10×10×0.5 mm) were degreased in acetone, stored in absolute ethanol and flamed prior to use as described previously [24]. The constituents of each metal tested are detailed in Table 1 and all were supplied by the Copper Development Association.

**Table 1:** Composition of metals used in the study.

Metal type	UNS^a^ no.	% composition					
		Cu	Zn	Sn	Ni	Fe	Cr
copper	C11000	100					
phosphor bronze (contains ∼ 0.26% P)	C51000	95		5			
copper nickel	C70600	89			10	1	
cartridge brass	C26000	70	30				
nickel silver	C75200	65	17		18		
stainless steel	S30400				8	74	18

a Unified Numbering System.

### Inoculation of metal coupons with MNV-1 (to simulate wet fomite or dry touch contamination) and assessment of infectious virus by the detection of cytopathic effect in murine cell line (plaque assay)

The surfaces of coupons were inoculated with MNV−1 5×10^4^ plaque forming units (pfu) in 20 µL or 5×10^4^ pfu in 1 µL to represent wet fomite (dries in 30–40 minutes at 22°C) or dry touch contamination (dries in seconds), respectively. Drying time was included in the exposure time. For temperatures other than ambient coupons were allowed to acclimatise for 30 minutes prior to inoculation. Some modifications were made to the method previously described for removing bacteria from coupons [24]. Viruses were removed from the coupons at the required timepoint by vortexing for 15 s (half the time for bacteria to reduce frothing) in 5 ml complete DMEM with approximately 100×2 mm diameter glass beads (twice the number used for bacteria). A range of dilutions was prepared immediately in complete DMEM and 1 mL aliquots were plated onto monolayers of RAW 264.7 that had been seeded with 10^6^ cells per well of 6 well plates (diameter 3.5 cm) 3 hours previously, and incubated at 37°C and 5% CO_2_ for 90 minutes. The inoculum was aspirated and overlay of 3 mL per well of 3% low melting point (LMP) agarose in complete medium was added to prevent virus spreading to other cells. Plates were incubated for 15 minutes at 4°C until set and then at 37°C, 5% CO_2_ for 72 hours. Monolayers were stained with 2 mL per well of a filtered 0.01% solution of the supravital stain, Neutral Red, which is pinocytosed by viable cells and accumulates in the cell lysosomes staining the cells red, in PBS for 2 hours at 37°C and 5% CO_2_. Excess stain was removed and the plates re-incubated for a further hour. Concentrations of stain > 100 µg/mL can be cytotoxic and should not be used. Plates were stored overnight at 4°C to increase definition of plaques which were counted and used to calculate pfu recovered per coupon.

The rate of virus inactivation was calculated for the following time periods: 0–5, 5–10 and 10–30 minutes for ‘dry’ inoculum and 0–30, 30–60 and 60–120 minutes for the ‘wet’ inoculum according to the following formula:



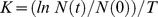





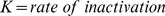





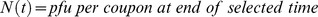





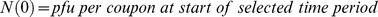





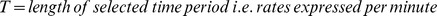



### The effect of copper chelators and reactive oxygen species quenchers in infectivity of MNV exposed to copper and copper alloy surfaces

Incorporation of chelators ethylenediaminetetraacetic acid (EDTA) (20 mM) and bathocuproine disulfonic acid (BCS) (20 mM) to chelate Cu(II) or Cu(I), respectively, at the time of inoculation of virus to the metal surfaces was investigated using plaque assay. In addition 20 mM D-mannitol and 20 mM 4,5-dihydroxy-1,3-benzene disulfonic acid (Tiron) were used to quench hydroxyl radicals and superoxide, respectively. Stainless steel was used as a control surface and to determine if quenchers and chelators affect viral replication.

### Survival of infectivity of MNV on metal surfaces at 37°C and 4°C

Metal surfaces were allowed to acclimatise to the test temperature for 30 minutes prior to inoculation with virus. Virus was removed from coupons and assessed for infectivity as described.

### Purification of viral RNA and analysis of integrity by agarose gel electrophoresis

The total RNA of untreated virus or virus exposed to metal surfaces (5 coupons per test, virus removed from coupons by pipetting up and down in a small volume, 100 µL) was extracted using the Qiagen QIAamp viral RNA mini kit according to manufacturer's instructions and using carrier RNA provided to prevent degradation.

Purified RNA fragments were separated on a non-denaturing 1% agarose gel using GelRed Nucleic Acid Prestaining Kit (Biotium, UK) according to the manufacturer's instructions. The staining intensity is reduced because GelRed binds to ssRNA approximately half as much as double stranded nucleic acid. DNA ladders were supplied by Bioline. Gels were observed and photographed using GeneSnap software and a Syngene UV light box.

### Detection and quantification of VPg in MNV exposed to copper and brass surfaces

cDNA was prepared from the purified viral RNA (RT-nanoscript) PrimerDesign, UK). Primers were designed to amplify a 70 bp region of VPg in ORF-1 from complete genome of MNV-1 CW1 (accession number DQ285629) (PrimerDesign Ltd., Southampton, UK)

sense primer GCGAGCGAGAAGAAGAACT (position 2761)

antisense primerTTCAACCCGAAGCCATCC (position 2380).

Amplification was performed on a BioRad iQ5 cycler and standard curves prepared from known copy number standards to determine copy number in test samples. A synthesised VPg cDNA was used to prepare standard curve and calculate copy number in equivalent volumes virus suspension applied to test surface samples. PCR products were analysed by gel electrophoresis as described.

### Statistical analysis

Data are expressed as mean ± standard errors of the mean (SEM) and are from multiple independent experiments. Differences between duplicate samples were assessed using the Mann-Whitney rank t-test. Group comparisons were analysed using the Mann-Whitney U test where statistical significance was expressed as p < 0.05. Statistical analyses and graphical representations were performed using Sigma Plot version 12.

## Supporting Information

Figure S1 **Linear regression analysis of virus inactivation rate (0–30 minutes, ‘wet’ inoculum) and percentage copper in alloys tested resulted in a coefficient of determination (R^2^) of 0.926 suggesting a good correlation.** The result for phosphor bronze was removed from this analysis. (including phosphor bronze reduced the R^2^ to 0.541). Further investigations into the efficacy of phosphor bronze to inactivate norovirus are planned including determining the influence of different metal surface finishes and other metal constituents.
(TIF)

Figure S2 **The entire RNA genome of untreated MNV (PEG concentrate) was purified as described in the text and fragments separated by electrophoresis on a non-denaturing 1% agarose gel (lane 2).** Lane 3 shows 18 s and 28 s cellular RNA from uninfected RAW 264.7 cells and lanes 1 and 4 are Bioline Hyperladders I and II, respectively.
(TIF)
