# Correction: Inactivation of Norovirus on Dry Copper Alloy Surfaces

**DOI:** 10.1371/journal.pone.0098333

**Published:** 2014-05-20

**Authors:** The PLOS ONE Staff

There is an error in the primer position under the sub-heading “Detection and quantification of VPg in MNV exposed to copper and brass surfaces” in the “Materials and Methods” section. The correct antisense primer position is TTCAACCCGAAGCCATCC (position 2830).

In addition, lanes 1 and 3 are labeled incorrectly in the legend for Figure 5. Please view the correct legend and figure here:

**Figure 5:**
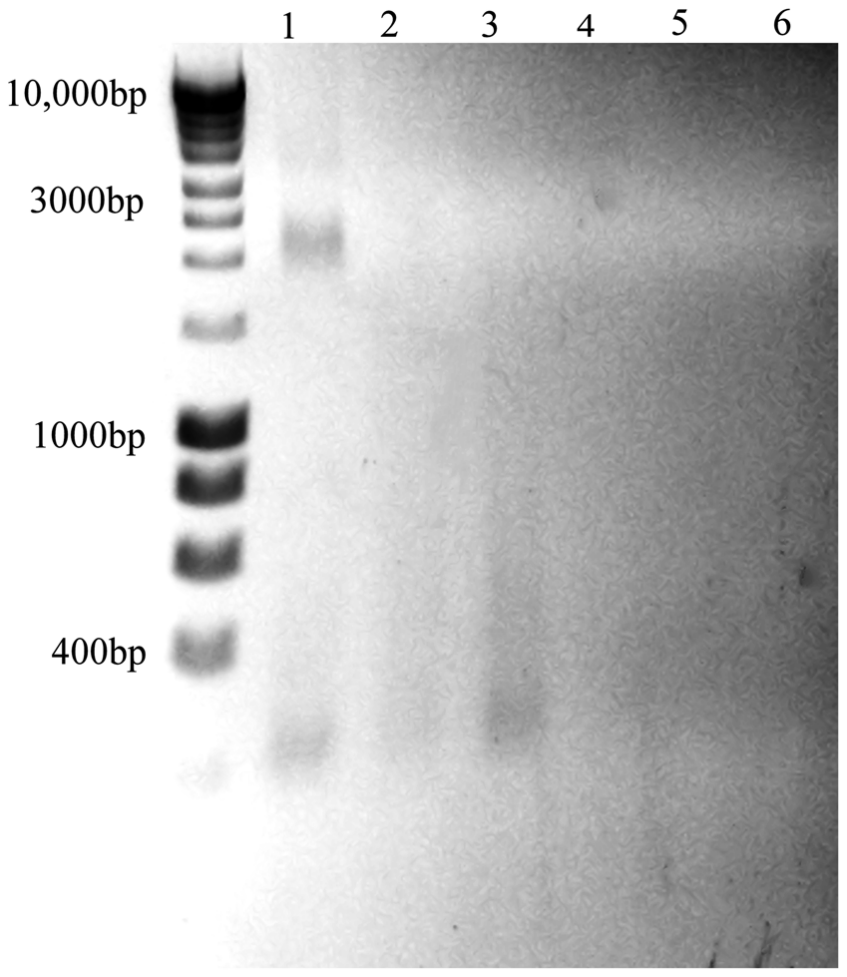
Destruction of entire MNV genome occurs on copper. MNV (PEG concentrate) was exposed to copper (lane 3), cartridge brass (lane 2) or stainless steel (lane 1) for 2 hours. Viral RNA was purified using Qiagen mini prep viral RNA kit and fragments separated on non-denaturing 1% agarose gel electrophoresis and visualised in UV light box. Viral RNA has degraded on copper, less on brass and not at all on stainless steel (see control RNA S2 Supplementary Information). Lanes 4, 5 and 6 are PEG precipitation of uninfected cells (mock) applied to stainless steel, brass and copper respectively. Virus added to all surfaces and removed immediately was similar to lane 1 although some reduction in intensity on copper was visible (not shown). DNA ladder is Bioline hyperladder I (HL1 1 Kb).
