# Supplementary figures and images for: Inactivation of Norovirus on Dry Copper Alloy Surfaces
Source: PLoS One. 2013 Sep 9;8(9):e75017. doi: 10.1371/journal.pone.0075017 (PMC3767632; doi:10.1371/journal.pone.0075017)

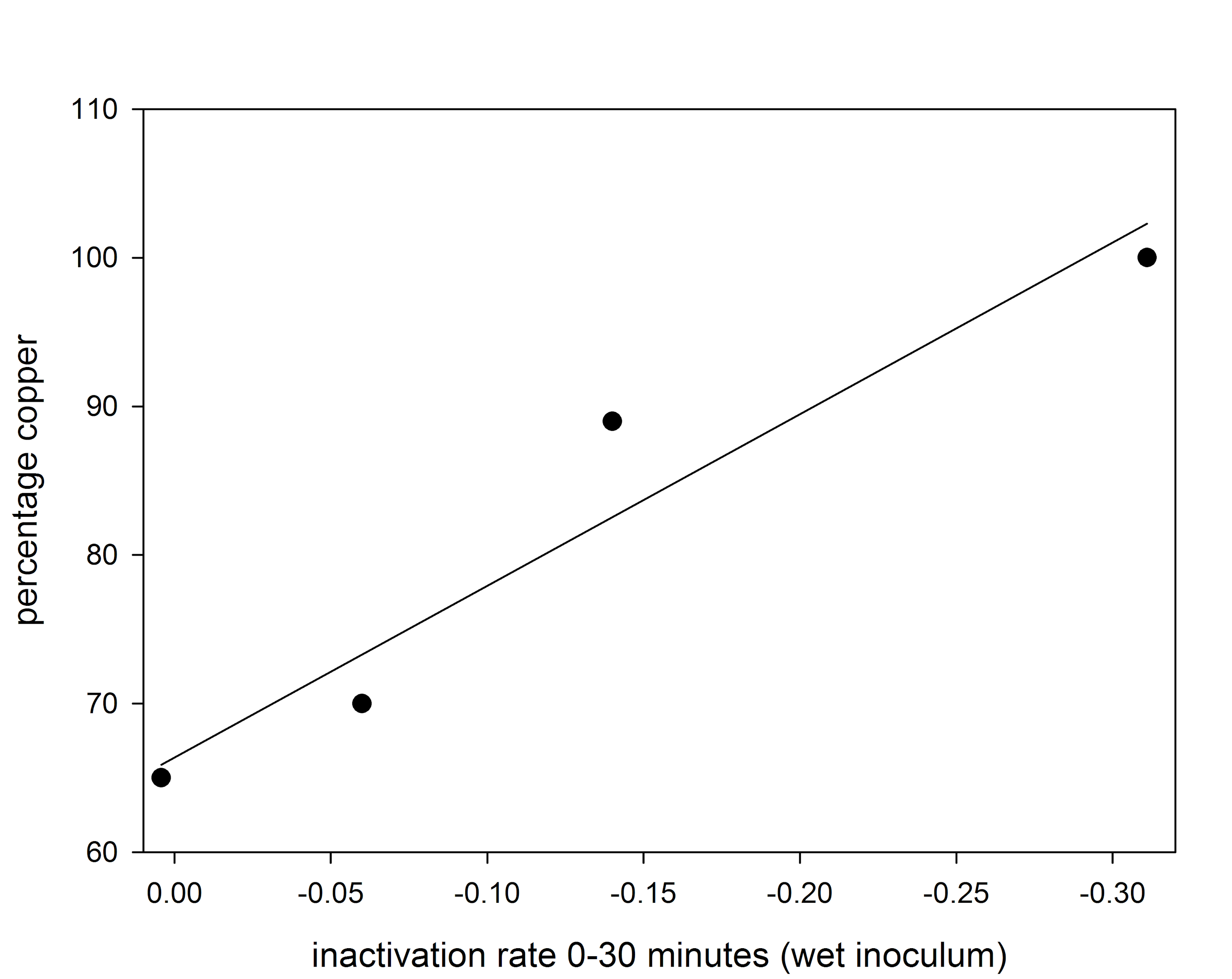

Supplement: Figure S1 — Linear regression analysis of virus inactivation rate (0–30 minutes, ‘wet’ inoculum) and percentage copper in alloys tested resulted in a coefficient of determination (R2) of 0.926 suggesting a good correlation. The result for phosphor bronze was removed from this analysis. (including phosphor bronze reduced the R2 to 0.541). Further investigations into the efficacy of phosphor bronze to inactivate norovirus are planned including determining the influence of different metal surface finishes and other metal constituents. (TIF) [file pone.0075017.s001.tif]

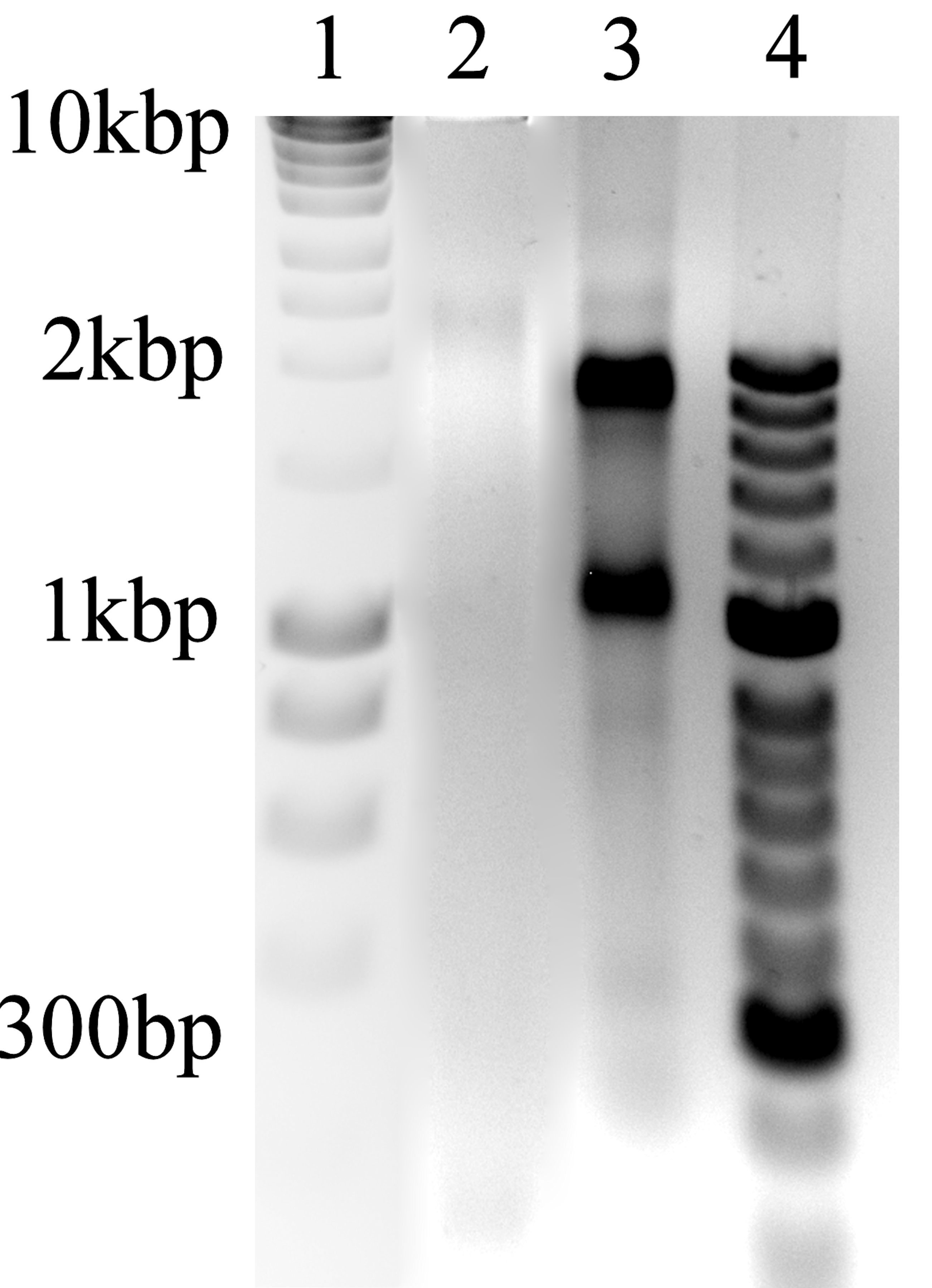

Supplement: Figure S2 — The entire RNA genome of untreated MNV (PEG concentrate) was purified as described in the text and fragments separated by electrophoresis on a non-denaturing 1% agarose gel (lane 2). Lane 3 shows 18 s and 28 s cellular RNA from uninfected RAW 264.7 cells and lanes 1 and 4 are Bioline Hyperladders I and II, respectively. (TIF) [file pone.0075017.s002.tif]
